# Supplementary material for: MAF1 is a chronic repressor of RNA polymerase III transcription in the mouse
Source: Sci Rep. 2020 Jul 20;10:11956. doi: 10.1038/s41598-020-68665-0 (PMC7371695; doi:10.1038/s41598-020-68665-0)
Supplement: Supplementary file 1 — Supplementary file1 (PDF 3651 kb) [file 41598_2020_68665_MOESM1_ESM.pdf]

SUPPLEMENTARY INFORMATION

MAF1 is a Chronic Repressor of RNA Polymerase III Transcription in the Mouse

Nicolas Bonhoure[1], Viviane Praz[1,2], Robyn D. Moir[3], Gilles Willemin[1], François Mange[1], Catherine Moret[1], Ian M. Willis[3,4]* and Nouria Hernandez[1]*

1 Center for Integrative Genomics, Faculty of Biology and Medicine, University of Lausanne, 1015 Lausanne, Switzerland

2 Swiss Institute of Bioinformatics, 1015 Lausanne, Switzerland

3 Department of Biochemistry, Albert Einstein College of Medicine, Bronx, New York 10461, USA

4 Department of Systems and Computational Biology, Albert Einstein College of Medicine, Bronx, New York 10461, USA

* Corresponding authors

List of supplementary items

Figure S1. Analysis of Pol III occupancy scores.

Figure S2. Rank-rank hypergeometric overlap heatmaps.

Figure S3. Polysome profiles and bioinformatic analysis of ribosome profiling data.

Full-size uncropped images of Figure 3A.

Full-size uncropped images of Figure 6D

Table S1. Pol III occupancy of the loci examined in this work. Related to Figures 1 and 2.

Table S2. Pol III Loci above the cut-off in at least one condition shown in Figure 1B.

Table S3. Pol III Loci above the cut-off in at least one condition shown in Figure 2B.

Table S4. Pol III Loci above the cut-off in at least one condition shown in Figure 2C.

Table S5. Pol III Loci above the cut-off in at least one condition shown in Figure 2D.

Table S6. RNA-seq analysis of fed liver from wild-type and Maf1-/- mice.

Table S7. GO bioprocess enrichment of differentially expressed genes in fed liver of wild-type

Maf1-/- mice.

Table S8. Ribosome footprinting of liver samples from wild-type Maf1-/- mice.

Table S9. Xtail analysis of ribosome footprinting data.

Isoacceptors_perl

Isotypes_perl

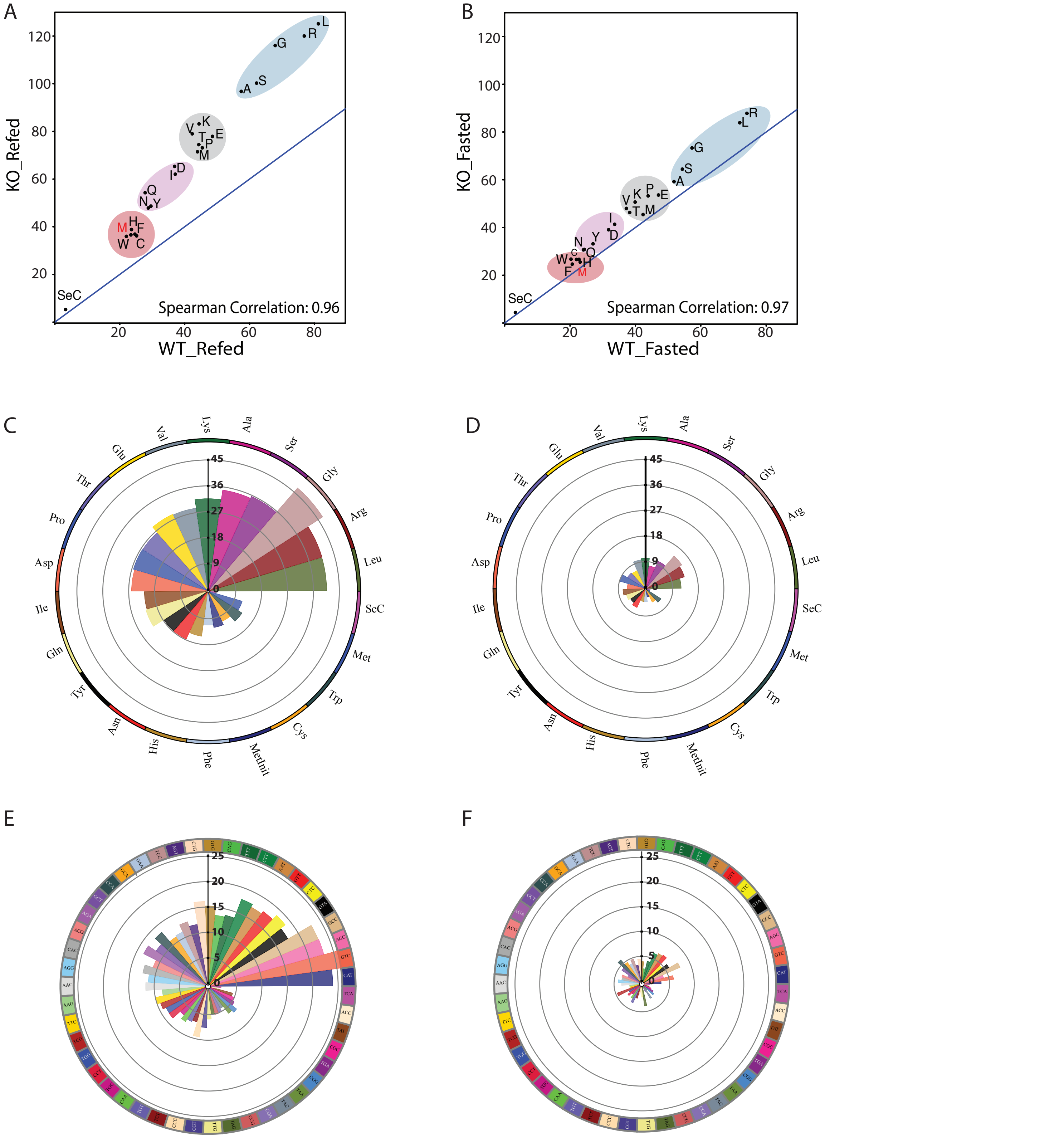

Figure S1. (A) Spearman rank correlations of pol III occupancy scores cumulated per isotype in WT and Maf1$^{-/-}$ mice in the refed condition. Twenty-one tRNA gene isotypes (tRNA^{SeC} gene not included and tRNAi^{Met} genes were considered separately from tRNA^{Met} genes) were clustered in four different quantiles (blue, grey, purple and pink ovals) according to their pol III occupancy. (B) As in panel A but for mice in the fasted condition. (C) Circular plot indicating the ratio of cumulated pol III occupancy scores between Maf1$^{-/-}$ and WT samples for each tRNA isotype in the refed condition. Isotypes were ordered according to cumulated scores in the WT sample. (D) As in panel C but for the 8 hour fasted condition. (E) Circular plot indicating the ratio of cumulated pol III occupancy scores between Maf1$^{-/-}$ and WT samples for each tRNA isoacceptor in the refed condition. Isoacceptors were ordered according to cumulated scores in the WT sample. (F) As in panel E but for the 8 hour fasted condition. Panels A and B were generated in R with the "plot" function from the R-base package. Additional highlights on quantiles were added by hand. Panels C-F are SVG images (http://www.w3.org/2000/svg) generated via in-house perl scripts "isoacceptor_perl" and "isotype_perl" and based on the RPC4 occupancy scores listed in Table S1.

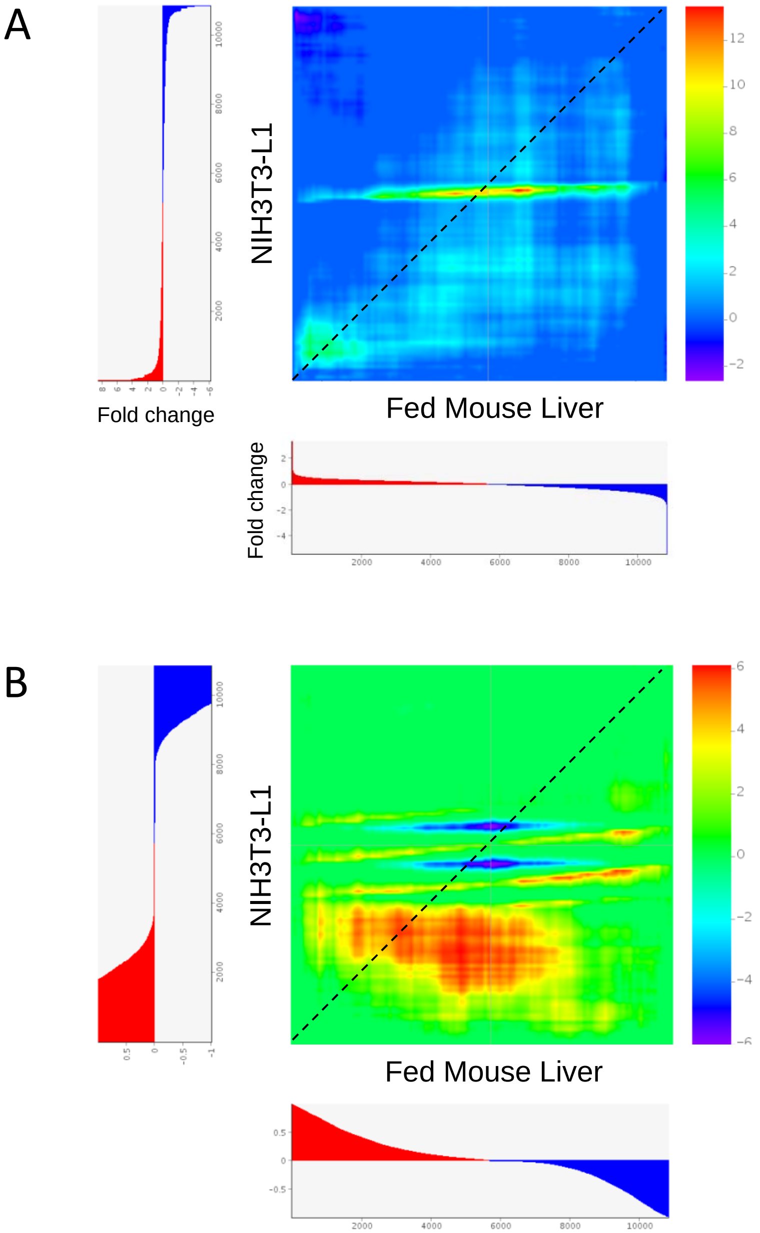

Figure S2. Rank-rank hypergeometric overlap heatmaps performed as described in Plaisier et al (2010)[25]. The software version is the original version, the link to the software is: http://systems.crump.ucla.edu/rankrank/. The effects of *Maf1* knockout or knockdown on gene expression are compared in mouse liver and undifferentiated NIH3T3-L1 cells, respectively. Overlaps were computed from a list of 10852 genes that were scored in both samples (this work Table S6 versus GEO dataset GSE113324 day zero samples before addition of differentiation cocktail). (A) Genes are ranked by fold change in gene expression. (B) Genes are ranked by adjusted log10 P values that are sign-adjusted depending on whether gene expression increased or decreased. In both panels, correlated gene expression patterns lie in proximity to the diagonal (dotted line) with upregulated genes in the bottom left quadrant and downregulated genes in the top right quadrant. The scale bar on the right shows the log10-transformed hypergeometric P values (Benjamini-Yekutieli corrected) with positive values indicating over-enrichment and negative values indicating under-enrichment. The metric values for each heatmap are plotted adjacent to the x- and y-axes and are colored red or blue to indicate increased or decreased values, respectively. Note that the highest hypergeometric P-values in both maps (red) correspond to gene ranks that are uncorrelated or correspond to fold changes or adjusted P values that are below thresholds for significance.

A

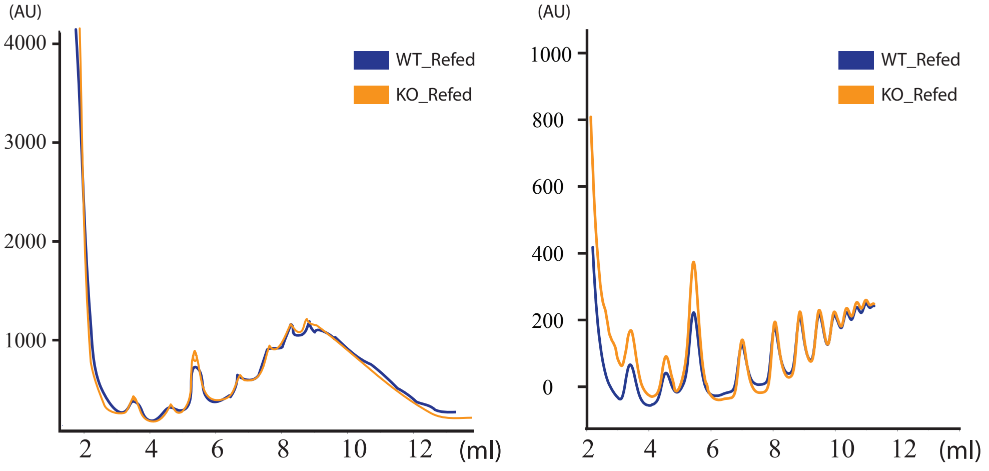

B

KEGG pathways	Adj. Pvalue
Ribosome	2.14 x10^{-05}
Ovarian steroidogenesis	0.0032
Proteoglycans in cancer	0.0060
Insulin secretion	0.0138
One carbon pool by folate	0.0162
Long term depression	0.0167

GO molecular functions	Adj. Pvalue
Structural constituent of ribosome	7.16 x10^{-05}
Protein binding	0.0029
Transcription factor activity	0.0058
Profilin binding	0.0059
ATP-dependent microtubule motor activity	0.0094
Phosphoprotein phosphatase activity	0.0122

Figure S3. A) Polysome profiles of liver samples from three WT (blue curves) and three Maf1$^{-/-}$ (orange curves) 22-24 week old fasted mice. (B) KEGG pathway[28-30] (left) (www.genome.jp/kegg/pathway.html) and GO molecular function (right) analysis for the genes showing lower translation efficiency (p-value <0.1) in the ribosome profiling experiment.

Northern blot images before cropping in Figure 3A, WAT panels.
Arrows indicate the annotated RNA species detected by sequential
hybridization with the indicated probes from left to right.

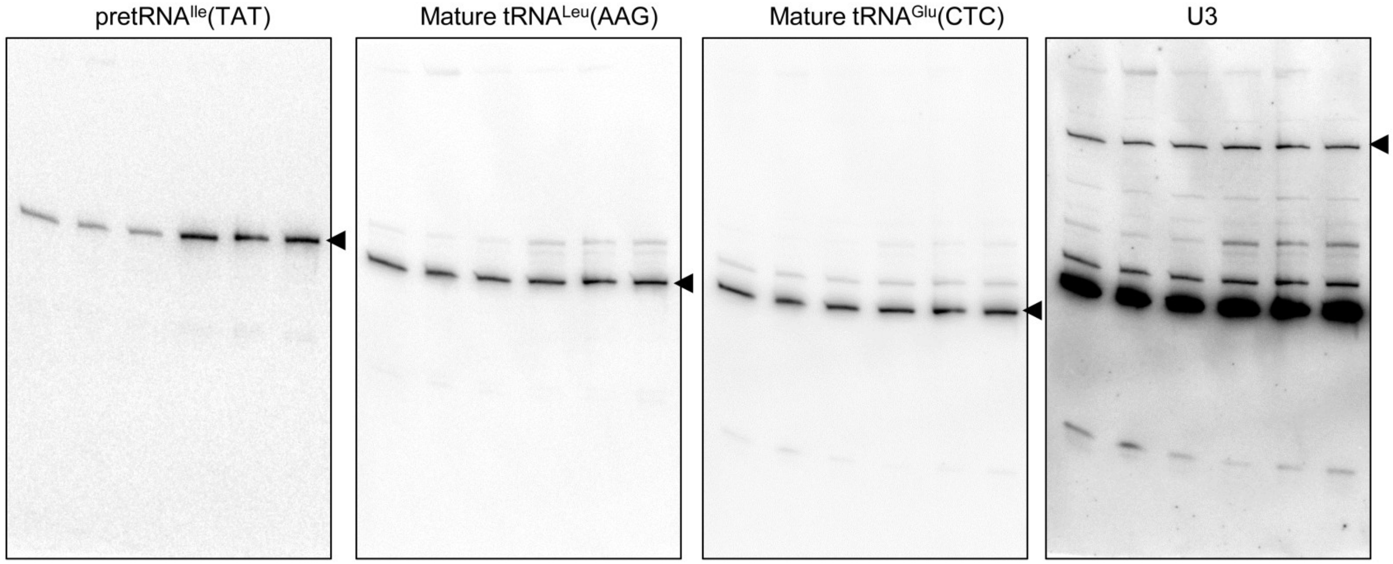

Northern blot images before cropping in Figure 3A, liver panels.
Arrows indicate the annotated RNA species
The blot was allowed to decay completely before hybridization with mature
tRNA probes, hence the absence of U3 and pretRNA^{Ile} signals

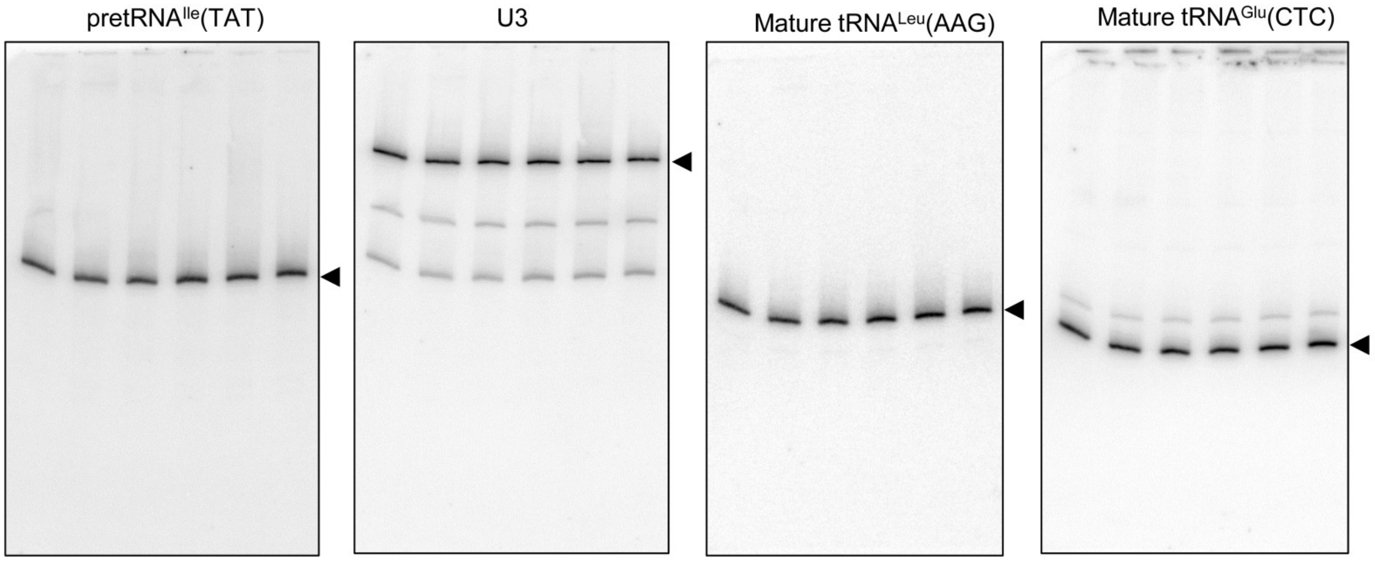

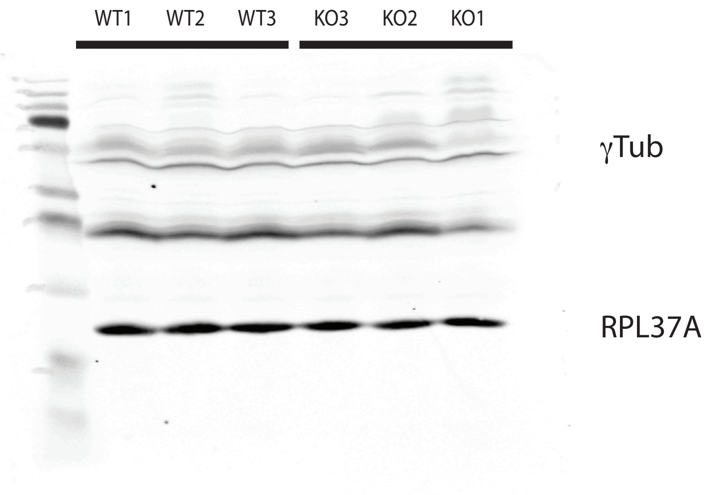

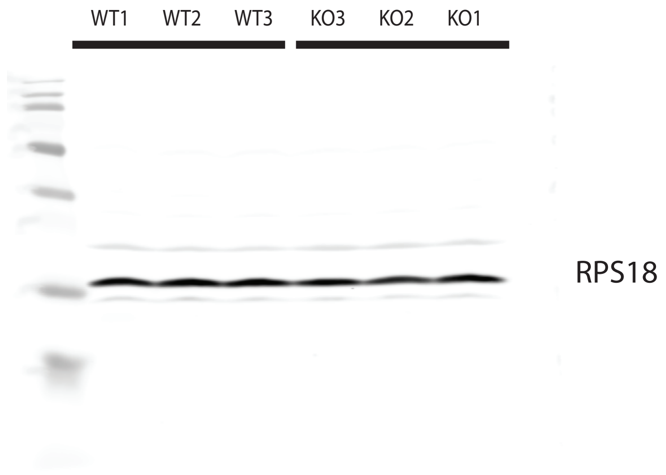

Full size immunoblots of liver lysates from WT or Maf1-/- mice performed with antibodies directed against the indicated proteins, before cropping for Figure 6D. Both images were acquired from the same gel using a different channel with the Li-COR system

```
#!/usr/bin/perl

### Input file for this script is a tab-delimited file which
contains the Mouse final scores for each experiment.
### The standard table is the output of the first R analysis, after
the data
# have been normalized to total tag count and scaled with human
spikes

### e.g. : /export/Nicolas/NEW_DATA/Tables/
Mouse_RPC4_scores_and_means_scaled_for_SVG.tab

### If the data column number is given as argument to the script,
the it takes the column.
### Otherwise, it will show the column titles and ask for the one to
use.


my $infile=$ARGV[0];
my $column_1=$ARGV[1];
my $column_2=$ARGV[2];

my $colorfile="/export/SVG/isotypes.txt";
# The color file contains 3 tab-delimited fields :
 # 1- isoacceptor
 # 2- isotype
 # 3- color

my %cutoff=(
    "R_WT_mean" => "1.6194",
    "R_KO_mean" => "1.8839",
    "F8H_WT_mean" => "0.6558",
    "F8H_KO_mean" => "0.5622"
    );

my %color=();
my %font_color=();
open(COL,"$colorfile");
while(<COL>)
{
    chomp;
    my @field=split/\t/;
    $color{$field[0]}=$field[2];
    $color{$field[1]}=$field[2];
    $font_color{$field[1]}=$field[3];
    $font_color{$field[0]}=$field[3];
}
close(COL);

my %sample=();

my $all_max=0;
open(MAX,"grep \'tRNA\' $infile | grep -v \'Sup\' |");
while(<MAX>)
```

```
{
    chomp;
    my @field=split/\t/;
    for(my $i=1;$i<scalar(@field);$i++)
    {
        if($field[$i]>$all_max)
        {
            $all_max=$field[$i];
        }
    }
}
close(MAX);


if(!$column)
{
    print "No column selected, extracting sample names from input
file\n\n";
}

open(IN,"head -1 $infile |");
while(<IN>)
{
    chomp;
    s/ /_/g;
    s/\(/_/g;
    s/\)/_/g;
    my $i=0;
    my @field=split/\t/;
    $sample{$i}=$fields[1];
    $col{$fields[0]}=$fields[1];

    foreach my $field(@field)
    {
        $sample{$i}=$field;
        if(!$column)
        {
            print "$i\t$field\n";
        }
        $i++;
    }
}
close(IN);
if(!$column_1)
{
    print "Select the first sample column number from the above
list :\n";
    chomp($column_1 = <STDIN>);
    print "Select the second sample column number from the above
list :\n";
    chomp($column_2 = <STDIN>);
}

my $title=$sample{$column_1};
```

```
$title.="_and_".
$sample{$column_2}."_ratio_by_isoacceptors_sorted_by_score_same_scal
e";
print "$title\n";
my $cutoff_1=$cutoff{$sample{$column_1}};
my $cutoff_2=$cutoff{$sample{$column_2}};
my $gene_num=0;
my %score_1=();
my %score_2=();
my %score_ref=();
my %ratio=();
my %isotype=();
my %isoacceptor=();
my @tRNAs=();
my %isoacceptor_count=();
my %isotype_count=();
my $max_score=0;
my $min_score=0;
my @acceptors=();
my @types=();
open(IN,"grep \'tRNA\' $infile | grep -v \'Sup\' |");
open(OUT,">$title.tmp");
while(<IN>)
{
    s/tRNA//;
    s/\-\|/\|/;
    s/\+\|/\|/;
    s/\(e\)//;
    chomp;
    my @field=split/\t/;
    my @name=split/\|/,$field[0];
    my @type=split/_/,$name[1];
#
if(($field[$column_1]<=$cutoff_1)&&($field[$column_2]<=$cutoff_2))
#    {
#        $field[$column_1]=0;
#        $field[$column_2]=0;
#    }
    if($field[$column_1]<0)
    {
        $field[$column_1]=0;
    }
    if($field[$column_2]<0)
    {
        $field[$column_2]=0;
    }
    my $rat=$field[$column_2]-$field[$column_1];
    $gene_num++;
    print OUT
("$name[0]\t$type[2]\t$type[3]\t$field[$column_1]\t$field[$column_2]
\t$rat\t$field[2]\n");
}
close(IN);
close(OUT);
```

```
my %code=();
my %count_by_type=();
my %count_by_iso=();

open(TMP,"$title.tmp");
while(<TMP>)
{
    chomp;
    my @field=split/\t/;
    push(@tRNAs,$field[0]);
    if(!$code{$field[1]}{$field[2]})
    {
        $count_by_type{$field[1]}++;
        $code{$field[1]}{$field[2]}=1;
    }
    if(!$isoacceptor_count{$field[2]})
    {
        $isoacceptor_count{$field[2]}=1;
        push(@acceptors,$field[2]);
    }
    else
    {
        $isoacceptor_count{$field[2]}++;
    }
    if(!$isotype_count{$field[1]})
    {
        $isotype_count{$field[1]}=1;
        push(@types,$field[1]);
    }
    else
    {
        $isotype_count{$field[1]}++;
    }
    $isotype{$field[2]}=$field[1];
    $score_1{$field[2]}+=$field[3];
    $score_2{$field[2]}+=$field[4];
    $ratio{$field[2]}+=$field[5];
    $score_ref{$field[2]}+=$field[6];
    if($ratio{$field[2]}<$min_score)
    {
        $min_score=$ratio{$field[2]};
    }
    if($ratio{$field[2]}>$max_score)
    {
        $max_score=$ratio{$field[2]};
    }
}
close(TMP);
open(OUT,">$title\_by_acceptor.tmp");
foreach my $acceptor(@acceptors)
{
    my $mean_score=int(($score_1{$acceptor}+$score_2{$acceptor})/2);
    print OUT ("$acceptor\t$score_1{$acceptor}\t$score_2{$acceptor}
\t$mean_score\t$score_ref{$acceptor}\n");
```

```
}
close(OUT);
system("sort -t \"\t\" -k 5,5nr $title\_by_acceptor.tmp >
$title\_by_acceptor.txt");

my @acceptors=();
open(ACCEPTORS,"$title\_by_acceptor.txt");
while(<ACCEPTORS>)
{
    chomp;
    my @f=split/\t/;
    push(@acceptors,$f[0]);
}
close(ACCEPTORS);
$max_score=25.5;
open(OUT,">$title.svg");
print "ALL max $all_max\nMAX score $max_score\nMIN score
$min_score\n";
print OUT ("   <!DOCTYPE HTML>\n<html xmlns=\"http://www.w3.org/
1999/xhtml\"><head>\n <title>$title pie chart</title>\n<style
type=\"text/css\" media=\"screen\">\nsvg {display:block; border:2px
solid #00c; position:relative;\ntop:0%; left:0%; width:100%; height:
100%; background:#fff;\n}\n</style>\n</head><body>\n<svg
width=\"1200\" height=\"1200\" viewBox=\"0 0 1200
1200\"\nxmlns=\"http://www.w3.org/2000/svg\" version=\"1.1\">\n");
my $angle_sum=0;
my $rad_angle_sum=0;
my $c=360;
my $acceptor_num=scalar(@acceptors);
my $total_length=abs($min_score)+0.6+abs($max_score);
foreach my $type(@types)
{
        my $split_val=(360/$acceptor_num)*$count_by_type{$type};
        $c-=$split_val;
        print "$type\t$count_by_type{$type}\n";
        my $type_angle = atan2(1,1) * $split_val / 45;
        my $text_angle=6.283185307-($rad_angle_sum+($type_angle/
2));
        my $length=460;
        my $length_1=450;
        my $textlength=480;
        my $x_textpos=(cos($text_angle) * $textlength)+600;
        my $y_textpos=(sin($text_angle) * $textlength)+600;
        my $first_line=$length-$length_1;
        my $start_pos=600+$length_1;
        my $xpos_2=(cos($type_angle) * $length)-$length;
        my $ypos_2=sin($type_angle) * $length;
        my $xpos_1=(cos($type_angle) * $length_1);
        my $ypos_1=sin($type_angle) * $length_1;
        my $xfinal=$length_1-$xpos_1;
        my $yfinal=0-$ypos_1;
        $xpos_1=$xpos_1-$xpos_2-$length;
        $ypos_1=$ypos_1-$ypos_2;
        my $first_turn=0;
```

```
        my $sec_turn=1;
        if($ypos_1<0)
        {
            $first_turn=1;
        }
        if($ypos_2>0)
        {
            $sec_turn=0;
        }
        my $rotation=$c;
        my $anchor="start";
        if(($c>90)&&($c<270))
        {
            $rotation=$c-180;
            $anchor="end";
        }
        print OUT ("<path id= \"$type\" d=\"M $start_pos,600 l
$first_line,0 a$length,$length 0 0,$first_turn $xpos_2,$ypos_2 l
$xpos_1,$ypos_1 a$length_1,$length_1 0 0,$sec_turn $xfinal,$yfinal
z\" stroke=\"black\" stroke-width=\"1\" fill=\"none\"
transform=\"rotate($c 600 600)\"\/>\n");
        print OUT ("<text x=\"$x_textpos\" y=\"$y_textpos\"
style=\"text-anchor: $anchor;font-family: Times New Roman;\"
fill=\"black\" transform=\"rotate($rotation $x_textpos,$y_textpos)
\">$type</text>\n");
#       print
"$x_textpos\t$y_textpos\t$c\t$rotation\t$text_angle\t$split_val\t$ty
pe\n";
        $rad_angle_sum+=$type_angle;
}

my $rad_angle_sum=0;
$c=360;

foreach my $acceptor(@acceptors)
{
        my $split_val=360/$acceptor_num;
        $c-=$split_val;
        my $acceptor_angle = atan2(1,1) * $split_val / 45;
        my $text_angle=6.283185307-($rad_angle_sum+
($acceptor_angle/2));
        my $length=445;
        my $length_1=405;
        my $textlength=410;
        my $x_textpos=(cos($text_angle) * $textlength)+600;
        my $y_textpos=(sin($text_angle) * $textlength)+600;
        my $first_line=$length-$length_1;
        my $start_pos=600+$length_1;
        my $xpos_2=(cos($acceptor_angle) * $length)-$length;
        my $ypos_2=sin($acceptor_angle) * $length;
        my $xpos_1=(cos($acceptor_angle) * $length_1);
        my $ypos_1=sin($acceptor_angle) * $length_1;
        my $xfinal=$length_1-$xpos_1;
        my $yfinal=0-$ypos_1;
```

```
        $xpos_1=$xpos_1-$xpos_2-$length;
        $ypos_1=$ypos_1-$ypos_2;
        my $first_turn=0;
        my $sec_turn=1;
        if($ypos_1<0)
        {
            $first_turn=1;
        }
        if($ypos_2>0)
        {
            $sec_turn=0;
        }
        my $rotation=$c;
        my $anchor="start";
        if(($c>90)&&($c<270))
        {
            $rotation=$c-180;
            $anchor="end";
        }
        print OUT ("<path id= \"$acceptor\" d=\"M $start_pos,600 l
$first_line,0 a$length,$length 0 0,$first_turn $xpos_2,$ypos_2 l
$xpos_1,$ypos_1 a$length_1,$length_1 0 0,$sec_turn $xfinal,$yfinal
z\" stroke=\"grey\" stroke-width=\"1\" fill=\"$color{$acceptor}\"
transform=\"rotate($c 600 600)\" \/>\n");

        print OUT ("<text x=\"$x_textpos\" y=\"$y_textpos\"
style=\"text-anchor: $anchor;font-family: Times New Roman;font-size:
10px;fill :$font_color{$acceptor}\" fill=\"black\"
transform=\"rotate($rotation $x_textpos,$y_textpos)\">$acceptor</
text>\n");
#       print OUT ("<text x=\"$x_textpos\" y=\"$y_textpos\"
textLength=\"30\" font-weight=\"1\" style=\"text-anchor: $anchor;
stroke : $fontcolor; font-weight : 1;font-family: Times New
Roman;font-size:1\" transform=\"rotate($rotation $x_textpos,
$y_textpos)\">$acceptor</text>\n");
        $rad_angle_sum+=$acceptor_angle;
}

my $angle = atan2(1,1) * (360/$acceptor_num) / 45;
my $degree_angle=360/$acceptor_num;
$c = 360;

my $count=0;
my $circle_r=((abs(int($min_score)-0.6)/$total_length)*400);
my $zero_pos=$circle_r+600;
my $zero_xpos=$zero_pos+((cos($angle) * $circle_r)-$circle_r);
my $zero_ypos=600+(sin($angle) * $circle_r);
print "$zero_pos\t$zero_xpos\t$zero_ypos\t$max_score\t$min_score\n";
print OUT ("<circle cx=\"600\" cy=\"600\" r=\"$circle_r\"
fill=\"none\" stroke=\"black\" stroke-width=\"1\"/>\n");

### Scores ###
print "Ratios\n";
foreach my $acceptor(@acceptors)
```

```
{
    $count++;
    $c -= $degree_angle;
    my $length_1=(($ratio{$acceptor})/$total_length)*400;
    print "$ratio{$acceptor}\n";
    my $xpos_1=((cos($angle) * $length_1)-$length_1);
    my $ypos_1=(sin($angle) * $length_1);
    if($ratio{$acceptor}<0)
    {
        print OUT ("<path id= \"$trna\" d=\"M $zero_pos,600 l
$length_1,0 a$length_1,$length_1 0 0,0 $ypos_1,$xpos_1 L $zero_xpos,
$zero_ypos z\"  style=\"stroke:$color{$acceptor};fill-opacity:
0.8;stroke-width:0.2;fill:$color{$acceptor}\" transform=\"rotate($c
600 600)\"\/>\n");
    }
    else
    {
    my $xpos_1=(cos($angle) * $length_1)-$length_1;
    my $ypos_1=sin($angle) * $length_1;
        print OUT ("<path id= \"$trna\" d=\"M $zero_pos,600 l
$length_1,0 a$length_1,$length_1 0 0,1 $xpos_1,$ypos_1 L $zero_xpos,
$zero_ypos z\"  style=\"stroke:$color{$acceptor};fill-opacity:
0.8;stroke-width:0.2;fill:$color{$acceptor}\" transform=\"rotate($c
600 600)\"\/>\n");
    }
}
my $step=int(($max_score-$min_score)/5);
print OUT ("<path d=\"M 600,600 l 0 -400\"
style=\"stroke:rgb(0,0,0);stroke-width:1\" />\n");
for(my $i=0;$i<$max_score;$i+=$step)
{
    my $length=($i/$total_length)*400;
    my $tick_pos=600-$circle_r-$length;
#    if($i<=0)
#    {
#        $tick_pos=600-$circle_r+$length;
#    }
    my $j=$i-int($i);
    if(($i<0)||(($i>=1)&&($j==0)))
    {
        my $ray=$circle_r+$length;
        print "$i\t$j\t$length\n";
        print OUT ("<circle cx=\"600\" cy=\"600\" r=\"$ray\"
fill=\"none\" stroke=\"grey\" stroke-width=\"0.5\"/>\n");
    }
    print OUT ("<path d=\"M 596 $tick_pos l 8 0\"
style=\"stroke:rgb(0,0,0);stroke-width:1\" />\n");
    print OUT ("<text x=\"602\" y=\"$tick_pos\" fill=\"black\"
style=\"text-anchor: start; stroke : #000000; font-size: 12px;font-
style: Times New Roman;text-length : 30;fill: none;\">$i</text>\n");

}
print OUT ("<text x=\"600\" y=\"50\" fill=\"black\" style=\"text-
anchor: middle; stroke : #000000; font-size: 30px;text-length :
```

```
30;fill: grey;\">$title</text>\n");
print OUT ("</svg>\n");
print OUT ("\n</body></html>\n");
close(OUT);
#system("/bin/rm $title.tmp $title.txt");
```

```
#!/usr/bin/perl

### Input file for this script is a tab-delimited file which
contains the Mouse final scores for each experiment.
### The standard table is the output of the first R analysis, after
the data
# have been normalized to total tag count and scaled with human
spikes

### e.g. : /export/Nicolas/NEW_DATA/Tables/
Mouse_RPC4_scores_and_means_scaled_for_SVG.tab

### If the data column number is given as argument to the script,
the it takes the column.
### Otherwise, it will show the column titles and ask for the one to
use.


my $infile=$ARGV[0];
my $column_1=$ARGV[1];
my $column_2=$ARGV[2];

my $colorfile="/export/SVG/isotypes.txt";
# The color file contains 3 tab-delimited fields :
 # 1- isoacceptor
 # 2- isotype
 # 3- color

my %cutoff=(
    "R_WT_mean" => "1.6194",
    "R_KO_mean" => "1.8839",
    "F8H_WT_mean" => "0.6558",
    "F8H_KO_mean" => "0.5622"
    );

my %color=();
my %font_color=();
open(COL,"$colorfile");
while(<COL>)
{
    chomp;
    my @field=split/\t/;
    $color{$field[0]}=$field[2];
    $color{$field[1]}=$field[2];
    $font_color{$field[1]}=$field[3];
    $font_color{$field[0]}=$field[3];
}
close(COL);

my %sample=();

my $all_max=0;
open(MAX,"grep \'tRNA\' $infile | grep -v \'Sup\' |");
while(<MAX>)
```

```
{
    chomp;
    my @field=split/\t/;
    for(my $i=1;$i<scalar(@field);$i++)
    {
        if($field[$i]>$all_max)
        {
            $all_max=$field[$i];
        }
    }
}
close(MAX);


if(!$column)
{
    print "No column selected, extracting sample names from input
file\n\n";
}

open(IN,"head -1 $infile |");
while(<IN>)
{
    chomp;
    s/ /_/g;
    s/\(/_/g;
    s/\)/_/g;
    my $i=0;
    my @field=split/\t/;
    $sample{$i}=$fields[1];
    $col{$fields[0]}=$fields[1];

    foreach my $field(@field)
    {
        $sample{$i}=$field;
        if(!$column)
        {
            print "$i\t$field\n";
        }
        $i++;
    }
}
close(IN);
if(!$column_1)
{
    print "Select the first sample column number from the above
list :\n";
    chomp($column_1 = <STDIN>);
    print "Select the second sample column number from the above
list :\n";
    chomp($column_2 = <STDIN>);
}

my $title=$sample{$column_1};
```

```
$title.="_and_".
$sample{$column_2}."_ratio_by_isotypes_sorted_by_score_same_scale";
print "$title\n";
my $cutoff_1=$cutoff{$sample{$column_1}};
my $cutoff_2=$cutoff{$sample{$column_2}};
my $gene_num=0;
my %score_1=();
my %score_2=();
my %score_ref=();
my %ratio=();
my %isotype=();
my %isoacceptor=();
my @tRNAs=();
my %isoacceptor_count=();
my %isotype_count=();
my $max_score=0;
my $min_score=0;
my @acceptors=();
my @types=();
open(IN,"grep \'tRNA\' $infile | grep -v \'Sup\' |");
open(OUT,">$title.tmp");
while(<IN>)
{
    s/tRNA//;
    s/\-\|/\|/;
    s/\+\|/\|/;
    s/\(e\)//;
    chomp;
    my @field=split/\t/;
    my @name=split/\|/,$field[0];
    my @type=split/_/,$name[1];
#
if(($field[$column_1]<=$cutoff_1)&&($field[$column_2]<=$cutoff_2))
#    {
#        $field[$column_1]=0;
#        $field[$column_2]=0;
#    }
    if($field[$column_1]<0)
    {
        $field[$column_1]=0;
    }
    if($field[$column_2]<0)
    {
        $field[$column_2]=0;
    }
    my $rat=$field[$column_2]-$field[$column_1];
    $gene_num++;
    print OUT
("$name[0]\t$type[2]\t$type[3]\t$field[$column_1]\t$field[$column_2]
\t$rat\t$field[2]\n");
}
close(IN);
close(OUT);
```

```
my %code=();
my %count_by_type=();
my %count_by_iso=();

open(TMP,"$title.tmp");
while(<TMP>)
{
    chomp;
    my @field=split/\t/;
    push(@tRNAs,$field[0]);
    if(!$code{$field[1]}{$field[2]})
    {
        $count_by_iso{$field[2]}++;
        $code{$field[1]}{$field[2]}=1;
    }
    if(!$isoacceptor_count{$field[2]})
    {
        $isoacceptor_count{$field[2]}=1;
        push(@acceptors,$field[2]);
    }
    else
    {
        $isoacceptor_count{$field[2]}++;
    }
    if(!$isotype_count{$field[1]})
    {
        $isotype_count{$field[1]}=1;
        push(@types,$field[1]);
    }
    else
    {
        $isotype_count{$field[1]}++;
    }
    $score_1{$field[1]}+=$field[3];
    $score_2{$field[1]}+=$field[4];
    $ratio{$field[1]}+=$field[5];
    $score_ref{$field[1]}+=$field[6];
    if($ratio{$field[1]}<$min_score)
    {
        $min_score=$ratio{$field[1]};
    }
    if($ratio{$field[1]}>$max_score)
    {
        $max_score=$ratio{$field[1]};
    }
}
close(TMP);
open(OUT,">$title\_by_type.tmp");
foreach my $type(@types)
{
    my $mean_score=int(($score_1{$type}+$score_2{$type})/2);
    print OUT ("$type\t$score_1{$type}\t$score_2{$type}
\t$mean_score\t$score_ref{$type}\n");
}
```

```
close(OUT);

$max_score=45.5;
system("sort -t \"\t\" -k 5,5nr $title\_by_type.tmp >
$title\_by_type.txt");
my @types=();
open(TYPES,"$title\_by_type.txt");
while(<TYPES>)
{
    chomp;
    my @f=split/\t/;
    push(@types,$f[0]);
}
close(TYPES);
open(OUT,">$title.svg");
print "ALL max $all_max\nMAX score $max_score\nMIN score
$min_score\n";
print OUT ("   <!DOCTYPE HTML>\n<html xmlns=\"http://www.w3.org/
1999/xhtml\"><head>\n <title>$title pie chart</title>\n<style
type=\"text/css\" media=\"screen\">\nsvg {display:block; border:2px
solid #00c; position:relative;\ntop:0%; left:0%; width:100%; height:
100%; background:#fff;\n}\n</style>\n</head><body>\n<svg
width=\"1200\" height=\"1200\" viewBox=\"0 0 1200
1200\"\nxmlns=\"http://www.w3.org/2000/svg\" version=\"1.1\">\n");
my $angle_sum=0;
my $rad_angle_sum=0;
my $c=360;
my $type_num=scalar(@types);
my $total_length=abs($min_score)+0.6+abs($max_score);
foreach my $type(@types)
{
        my $split_val=(360/$type_num);
        $c-=$split_val;
        print "$type\t$count_by_type{$type}\n";
        my $type_angle = atan2(1,1) * $split_val / 45;
        my $text_angle=6.283185307-($rad_angle_sum+($type_angle/
2));
        my $length=460;
        my $length_1=450;
        my $textlength=480;
        my $x_textpos=(cos($text_angle) * $textlength)+600;
        my $y_textpos=(sin($text_angle) * $textlength)+600;
        my $first_line=$length-$length_1;
        my $start_pos=600+$length_1;
        my $xpos_2=(cos($type_angle) * $length)-$length;
        my $ypos_2=sin($type_angle) * $length;
        my $xpos_1=(cos($type_angle) * $length_1);
        my $ypos_1=sin($type_angle) * $length_1;
        my $xfinal=$length_1-$xpos_1;
        my $yfinal=0-$ypos_1;
        $xpos_1=$xpos_1-$xpos_2-$length;
        $ypos_1=$ypos_1-$ypos_2;
        my $first_turn=0;
        my $sec_turn=1;
```

```
        if($ypos_1<0)
        {
            $first_turn=1;
        }
        if($ypos_2>0)
        {
            $sec_turn=0;
        }
        my $rotation=$c;
        my $anchor="start";
        if(($c>90)&&($c<270))
        {
            $rotation=$c-180;
            $anchor="end";
        }
        print OUT ("<path id= \"$type\" d=\"M $start_pos,600 l
$first_line,0 a$length,$length 0 0,$first_turn $xpos_2,$ypos_2 l
$xpos_1,$ypos_1 a$length_1,$length_1 0 0,$sec_turn $xfinal,$yfinal
z\" stroke=\"black\" stroke-width=\"1\" fill=\"$color{$type}\"
transform=\"rotate($c 600 600)\"\/>\n");
        print OUT ("<text x=\"$x_textpos\" y=\"$y_textpos\"
style=\"text-anchor: $anchor;font-family: Times New Roman;\"
fill=\"black\" transform=\"rotate($rotation $x_textpos,$y_textpos)
\">$type</text>\n");
#       print
"$x_textpos\t$y_textpos\t$c\t$rotation\t$text_angle\t$split_val\t$ty
pe\n";
        $rad_angle_sum+=$type_angle;
}

#my $rad_angle_sum=0;
#$c=360;
#
#foreach my $acceptor(@acceptors)
#{
#       my $split_val=360/$acceptor_num;
#       $c-=$split_val;
#       my $acceptor_angle = atan2(1,1) * $split_val / 45;
#       my $text_angle=6.283185307-($rad_angle_sum+
($acceptor_angle/2));
#       my $length=445;
#       my $length_1=405;
#       my $textlength=410;
#       my $x_textpos=(cos($text_angle) * $textlength)+600;
#       my $y_textpos=(sin($text_angle) * $textlength)+600;
#       my $first_line=$length-$length_1;
#       my $start_pos=600+$length_1;
#       my $xpos_2=(cos($acceptor_angle) * $length)-$length;
#       my $ypos_2=sin($acceptor_angle) * $length;
#       my $xpos_1=(cos($acceptor_angle) * $length_1);
#       my $ypos_1=sin($acceptor_angle) * $length_1;
#       my $xfinal=$length_1-$xpos_1;
#       my $yfinal=0-$ypos_1;
#       $xpos_1=$xpos_1-$xpos_2-$length;
```

```
#       $ypos_1=$ypos_1-$ypos_2;
#       my $first_turn=0;
#       my $sec_turn=1;
#       if($ypos_1<0)
#       {
#           $first_turn=1;
#       }
#       if($ypos_2>0)
#       {
#           $sec_turn=0;
#       }
#       my $rotation=$c;
#       my $anchor="start";
#       if(($c>90)&&($c<270))
#       {
#           $rotation=$c-180;
#           $anchor="end";
#       }
#       print OUT ("<path id= \"$acceptor\" d=\"M $start_pos,600 l
$first_line,0 a$length,$length 0 0,$first_turn $xpos_2,$ypos_2 l
$xpos_1,$ypos_1 a$length_1,$length_1 0 0,$sec_turn $xfinal,$yfinal
z\" stroke=\"grey\" stroke-width=\"1\" fill=\"$color{$acceptor}\"
transform=\"rotate($c 600 600)\" \/>\n");
#
#       print OUT ("<text x=\"$x_textpos\" y=\"$y_textpos\"
style=\"text-anchor: $anchor;font-family: Times New Roman;font-size:
10px;fill :$font_color{$acceptor}\" fill=\"black\"
transform=\"rotate($rotation $x_textpos,$y_textpos)\">$acceptor</
text>\n");
##      print OUT ("<text x=\"$x_textpos\" y=\"$y_textpos\"
textLength=\"30\" font-weight=\"1\" style=\"text-anchor: $anchor;
stroke : $fontcolor; font-weight : 1;font-family: Times New
Roman;font-size:1\" transform=\"rotate($rotation $x_textpos,
$y_textpos)\">$acceptor</text>\n");
#       $rad_angle_sum+=$acceptor_angle;
#}
#
my $angle = atan2(1,1) * (360/$type_num) / 45;
my $degree_angle=360/$type_num;
$c = 360;

my $count=0;
my $circle_r=((abs(int($min_score)-0.6)/$total_length)*400);
my $zero_pos=$circle_r+600;
my $zero_xpos=$zero_pos+((cos($angle) * $circle_r)-$circle_r);
my $zero_ypos=600+(sin($angle) * $circle_r);
print "$zero_pos\t$zero_xpos\t$zero_ypos\t$max_score\t$min_score\n";
print OUT ("<circle cx=\"600\" cy=\"600\" r=\"$circle_r\"
fill=\"none\" stroke=\"black\" stroke-width=\"1\"/>\n");

### Scores ###
print "Ratios\n";
foreach my $type(@types)
{
```

```
    $count++;
    $c -= $degree_angle;
    my $length_1=(($ratio{$type})/$total_length)*400;
    print "$ratio{$type}\n";
    my $xpos_1=((cos($angle) * $length_1)-$length_1);
    my $ypos_1=(sin($angle) * $length_1);
    if($ratio{$type}<0)
    {
         print OUT ("<path id= \"$trna\" d=\"M $zero_pos,600 l
$length_1,0 a$length_1,$length_1 0 0,0 $ypos_1,$xpos_1 L $zero_xpos,
$zero_ypos z\"  style=\"stroke:$color{$type};fill-opacity:
0.8;stroke-width:0.2;fill:$color{$type}\" transform=\"rotate($c 600
600)\"\/>\n");
    }
    else
    {
    my $xpos_1=(cos($angle) * $length_1)-$length_1;
    my $ypos_1=sin($angle) * $length_1;
         print OUT ("<path id= \"$trna\" d=\"M $zero_pos,600 l
$length_1,0 a$length_1,$length_1 0 0,1 $xpos_1,$ypos_1 L $zero_xpos,
$zero_ypos z\"  style=\"stroke:$color{$type};fill-opacity:
0.8;stroke-width:0.2;fill:$color{$type}\" transform=\"rotate($c 600
600)\"\/>\n");
    }
}
my $step=int(($max_score-$min_score)/5);
print OUT ("<path d=\"M 600,600 l 0 -400\"
style=\"stroke:rgb(0,0,0);stroke-width:1\" />\n");
for(my $i=0;$i<$max_score;$i+=$step)
{
    my $length=($i/$total_length)*400;
    my $tick_pos=600-$circle_r-$length;
#     if($i<=0)
#     {
#         $tick_pos=600-$circle_r+$length;
#     }
    my $j=$i-int($i);
    if(($i<0)||(($i>=1)&&($j==0)))
    {
         my $ray=$circle_r+$length;
         print "$i\t$j\t$length\n";
         print OUT ("<circle cx=\"600\" cy=\"600\" r=\"$ray\"
fill=\"none\" stroke=\"grey\" stroke-width=\"0.5\"/>\n");
    }
    print OUT ("<path d=\"M 596 $tick_pos l 8 0\"
style=\"stroke:rgb(0,0,0);stroke-width:1\" />\n");
    print OUT ("<text x=\"602\" y=\"$tick_pos\" fill=\"black\"
style=\"text-anchor: start; stroke : #000000; font-size: 12px;font-
style: Times New Roman;text-length : 30;fill: none;\">$i</text>\n");

}
print OUT ("<text x=\"600\" y=\"50\" fill=\"black\" style=\"text-
anchor: middle; stroke : #000000; font-size: 30px;text-length :
30;fill: grey;\">$title</text>\n");
```

```
print OUT ("</svg>\n");
print OUT ("\n</body></html>\n");
close(OUT);
#system("/bin/rm $title.tmp $title.txt");
```
